# Lung Ultrasound in Pediatric Acute Respiratory Distress Syndrome Received Extracorporeal Membrane Oxygenation: A Prospective Cohort Study

**DOI:** 10.3389/fped.2022.798855

**Published:** 2022-03-28

**Authors:** Yucai Zhang, Chunxia Wang, Fei Wang, Jingyi Shi, Jiaying Dou, Yijun Shan, Ting Sun, Yiping Zhou

**Affiliations:** ^1^Department of Critical Care Medicine, Shanghai Children’s Hospital, Shanghai Jiao Tong University, Shanghai, China; ^2^Institute of Pediatric Critical Care, Shanghai Jiao Tong University, Shanghai, China; ^3^Clinical Research Unit, Shanghai Children’s Hospital, Shanghai Jiao Tong University, Shanghai, China

**Keywords:** lung ultrasound (LUS) score, acute respiratory distress syndrome, extracorporeal membrane oxygenation, prognosis, children

## Abstract

**Objective:**

The aim of this study was to assess the prognostic value of the lung ultrasound (LUS) score in patients with pediatric acute respiratory distress syndrome (pARDS) who received extracorporeal membrane oxygenation (ECMO).

**Methods:**

A prospective cohort study was conducted in a pediatric intensive care unit (PICU) of a tertiary hospital from January 2016 to June 2021. The severe pARDS patients who received ECMO were enrolled in this study. LUS score was measured at initiation of ECMO (LUS-0 h), then at 24 h (LUS-24 h), 48 h (LUS-48 h), and 72 h (LUS-72 h) during ECMO, and when weaned from ECMO (LUS-wean). The value of LUS scores at the first 3 days of ECMO as a prognostic predictor was analyzed.

**Results:**

Twenty-nine children with severe pARDS who received ECMO were enrolled with a median age of 26 (IQR 9, 79) months. The median duration of ECMO support was 162 (IQR 86, 273) h and the PICU mortality was 31.0% (9/29). The values of LUS-72 h and LUS-wean were significantly lower in survivors than that in non-survivors (both *P* < 0.001). Daily fluid balance volume during the first 3 days of ECMO support were strongly correlated with LUS score [1st day: *r* = 0.460, *P* = 0.014; 2nd day: *r* = 0.540, *P* = 0.003; 3rd day: *r* = 0.589, *P* = 0.001]. The AUC of LUS-72 h for predicting PICU mortality in these patients was 1.000, and the cutoff value of LUS-72 h was 24 with a sensitivity of 100.0% and a specificity of 100.0%. Furthermore, patients were stratified in two groups of LUS-72 h ≥ 24 and LUS-72 h < 24. Consistently, PICU mortality, length of PICU stay, ratio of shock, vasoactive index score value, and the need for continuous renal replacement therapy were significantly higher in the group of LUS-72 h ≥ 24 than in the group of LUS-72 h < 24 (all *P* < 0.05).

**Conclusion:**

Lung ultrasound score is a promising tool for predicting the prognosis in patients with ARDS under ECMO support. Moreover, LUS-72 h ≥ 24 is associated with high risk of PICU mortality in patients with pARDS who received ECMO.

## Introduction

The mortality of severe acute respiratory distress syndrome (ARDS) patients was 33% (95% *CI*: 26–41) according to an international prospective study covering 145 pediatric intensive care units (PICUs) from 27 countries (1). The Pediatric Acute Lung Injury Consensus Conference (PALICC) recommended that extracorporeal membrane oxygenation (ECMO) should be considered to support children with severe pediatric ARDS (pARDS) when lung protective strategies result in inadequate gas exchange (2). Until now, ECMO has become increasingly usual for severe pARDS due to improvement in technology and deeper understanding about indications and contraindications (3, 4). However, it is still challenging to optimize management of ECMO, which is critical for reducing adverse events and ECMO-related complications. Developing convenient and non-invasive tools and methods will lead to a promising view for delicacy management of ECMO.

Extravascular lung water (EVLW) identified in patients with non-cardiogenic pulmonary edema reflects the severity of ARDS and could be detected by a pulse indicator continuous cardiac output (PiCCO_2_), but it is not convenient for pediatric patients (5, 6). Besides, lung computed tomography (CT) is regarded as the gold standard for non-invasive evaluation of pulmonary edema; however, exposure to ionizing radiation and transportation of the critically ill limit its application, especially in children (7). Lung ultrasound (LUS) is a non-invasive, radiation-free, and bedside tool for management of the critically ill. LUS score was proved to be an alternative method for monitoring pulmonary edema and may guide management strategies for ARDS in both adults and children (8–10). Moreover, LUS score or the number of LUS B-lines predicts the outcomes in acute heart failure or interstitial lung disease (11, 12). Until now, the value of LUS score in predicting the prognosis in patients with pARDS under ECMO support has not been systematically studied.

In this study, children with pARDS who received ECMO were prospectively enrolled, and LUS score was determined before and during ECMO support. We aimed to evaluate the potential value of LUS score as an accurate and easy method to predict the prognosis of these children with ECMO support.

## Materials and Methods

### Study Design and Patient Populations

A prospective cohort study was conducted in a 36-bed PICU of a tertiary-level teaching hospital (Shanghai Children’s Hospital, Shanghai Jiao Tong University, China). Our study was approved by the Ethics Committee of the hospital (Approval number: 2016R011-E02). All patients’ parents or relatives signed informed consent and agreed with ECMO support and LUS measurement, as well as enrolling patients in this study. Our ECMO center is one of the major ECMO centers for children in China, which has been registered at the Extracorporeal Life Support Organization (ELSO) (No. 663).

**FIGURE 1:**
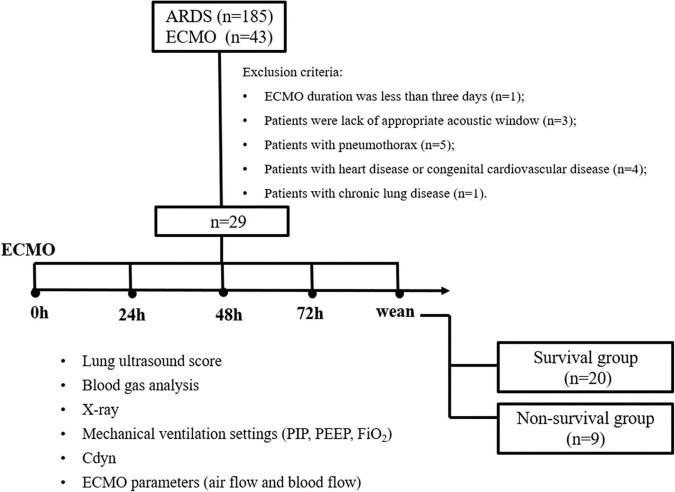
Flowchart of patients’ enrollment.

Patients with pARDS who received ECMO during PICU hospitalization were enrolled from January 2016 to June 2021. The pARDS is defined according to the 2015 PALICC definition of ARDS (2). Inclusion criteria included: (1) aged over 28 days to 14 years; and (2) the interval time between ARDS diagnosis and ECMO initiation less than 7 days. Exclusion criteria included: (1) ECMO duration was less than 3 days; (2) lack of appropriate acoustic window for LUS determination; (3) complicated with pneumothorax; (4) complicated with congenital heart disease; and (5) complicated with chronic lung disease (Figure 1). Patients with ARDS complicated with pneumothorax or chronic lung disease could receive ECMO as a rescued therapy. However, pneumothorax or chronic lung disease would lead to inaccurate measures about the LUS score, so these patients were excluded in this study.

### Data Collection

Demographics, comorbidities, complications, and outcomes were collected according to a pre-designed case report form (CRF). Demographics data included age, gender, and body mass index (BMI). The pediatric risk mortality III (PRISM III) score and comorbidities were determined at ECMO initiation. Clinical parameters included oxygen in inspired gas (FiO_2_), PaO_2_/FiO_2_ (PF ratio), oxygen index (OI), PaCO_2_, and Cdyn, which was calculated by ventilators (MAQUET Company, Servo-i serious), required ECMO blood flow and gas flow. The outcomes included PICU mortality, length of PICU stay, duration of ECMO, duration of ventilation, complications, and adverse events related to ECMO support.

### Ventilation Before Extracorporeal Membrane Oxygenation

Ventilation settings after patients meeting the diagnosis of pARDS complied with lung protective ventilation strategy, were positive end-expiratory pressure (PEEP) levels of 8–15 cmH_2_O and positive inspiration pressure (PIP) levels based on target tidal volume (Vt) of 4–8 ml/kg but less than 35 cmH_2_O (2).

### Extracorporeal Membrane Oxygenation and Mechanical Ventilation

Extracorporeal membrane oxygenation should be considered to support children with severe pARDS in whom the cause of the respiratory failure is reversible, or who is likely to be suitable for lung transplantation, or when lung protective strategies result in inadequate gas exchange; otherwise, ECMO should not be deployed in patients in whom life-sustaining measures are likely to be limited (13). In our center, we also referred to the adult-related ECMO indications [according to the guidelines from the ELSO Website outlined (14)] including: (1) PF ratio < 60–80 mmHg or OI > 40; (2) mean airway pressure (MAP) > 20–25 cmH_2_O on conventional ventilation; (3) evidence of iatrogenic barotrauma; (4) acute unremitting hypercapnic or hypoxic respiratory failure; (5) air leak syndrome; (6) mediastinal masses; and (7) cardiac failure. The contraindications for ECMO are (14): (1) lethal chromosomal abnormalities (trisomy 13 or 18); (2) severe neurologic compromise (intracranial hemorrhage with mass effect); and (3) incurable malignancy. The ECMO mode (ECMO machine: rotaflow, MAQUET Cardiopulmonary GmbH, Germany) included veno-arterial (VA) ECMO for complication with cardiac failure or hemodynamically unstable, and veno-venous (VV) ECMO for respiratory failure using a membrane lung. While native lung function supports adequate ventilation and oxygenation, weaning from VV-ECMO in patients requires cessation of gas-exchange support across the membrane oxygenator; and if adequate ventilation, oxygenation, and hemodynamic stability can be attained with a trial at low flow or no flow, patients could be separated from the VA-ECMO circuit (13). The adult-related indications for ECMO weaning were used as a reference in our center which included the following (14): The ABG was normal when fractional concentration of FiO_2_ was less than 0.5, PIP was less than 25 cmH_2_O, Cdyn was greater than 0.5 ml/kg.cmH_2_O, and required blood flow was less than 10 ml/kg, and required gas flow had been closed for more than 6 h.

The catheterization for ECMO was usually determined by the body weight of patients. In the present study, the VV-ECMO cannulae (Medtronic Bio-Medicus) were inserted through the right internal jugular vein and right femoral vein. For VA-ECMO, cannulae (Medtronic Bio-Medicus) were inserted through the right femoral artery and the right femoral vein in children with body weight over or equal to 15 kg; and ECMO cannulae (Medtronic Bio-Medicus) were inserted through the right internal carotid artery and right internal jugular vein through surgical incisions if the body weight of the patient was less than 15 kg.

Ventilation parameters during ECMO support according to the suggestion from ELSO guidelines (14) included low rate, long inspiratory time, PIP less than 25 cmH_2_O, FiO_2_ less than 0.4, and PEEP set at an appropriate level for patient condition.

### Lung Ultrasound Score

Lung ultrasound score was measured at the initiation of ECMO as the value of LUS-0 h. Then, the values were measured every morning in the following 3 days and at the termination of ECMO as LUS-24 h, LUS-48 h, LUS-72 h, and LUS-wean, respectively (Figure 1). For each of the LUS scores determined, corresponding ventilator and ECMO parameters were also recorded. LUS was performed by a PICU attending physician (independent scorer) who underwent a standard training. A 13–6 MHz curvilinear probe perpendicularly on the chest wall checks all intercostal spaces clearly (ultrasonography machine: M-Turbo Ultrasound System, Mini-Dock-M Series, SonoSite). Each hemithorax was divided into anterior, lateral, and posterior region according to the sternum, paravertebral, anterior, and posterior axillary lines, and each region was divided into superior and inferior halves (Supplementary Figure 1). All intercostal spaces of 12 areas were examined sequentially in supine, lateral, and prone positions. Each image should identify pleura lines and A-line. The scoring system in the present study is as described by Brat et al. (15).

### Statistical Analysis

All data were analyzed using SPSS 17.0 statistics (SPSS Inc., Chicago, IL, United States). Univariate comparisons of proportions were compared with chi-square tests. The continuous data with non-parametric distribution were expressed as median (interquartile range, IQR) and compared using the Mann-Whitney *U* test. The Friedman ANOVA was used to compare LUS score and Cdyn at 5 different time points during ECMO support (0 h, 24 h, 48 h, 72 h, and wean). The correlation between LUS score and Cdyn, and the correlation between the change of LUS scores and the change of daily fluid balance during the first 3 days after ECMO initiation were performed using a Bonferroni correction. All *P* values were two-sided and statistical significance was taken as *P* < 0.05.

## Results

### Baseline Characteristics of Patients With Pediatric Acute Respiratory Distress Syndrome Who Received Extracorporeal Membrane Oxygenation

A total of 185 patients with moderate to severe pARDS were admitted to a PICU from January 2016 to June 2021. Among them, 43 cases with severe pARDS received ECMO. According to the exclusion criteria, 1 case with less than 3 days ECMO duration, 3 cases with lack of appropriate acoustic window, 5 cases with pneumothorax, 4 cases with congenital heart disease, and 1 case with chronic lung disease were excluded. Finally, 29 patients were ultimately entered into analysis in this study. The median age of these patients was 26 (IQR, 9–79) months, and there were 14 male patients (48.3%, 14/29). Among 29 patients, 20 children survived and 9 children died. There were no significant differences in aspects of age, gender, PRISM III score, respiratory parameters, ventilator settings, and mode of ECMO between survivors and non-survivors (all *P* > 0.05) (Table 1).

**TABLE 1:** Baseline characteristics of patients at initiation of ECMO support.

Characteristics	Total (*n* = 29)	Survivors (*n* = 20)	Non-survivors (*n* = 9)	*P-value*
Age, month, IQR	26 (9–79)	37 (12–72)	15 (4–91)	0.383
Male, *n* (%)	14 (48.3)	8 (40.0)	6 (66.7)	0.184
PRISM III	18 (14–22)	17 (13–21)	25 (14–35)	0.093
BMI, kg/m^2^	14 (13–16)	14 (13–15)	16 (14–19)	0.109
Lactate, mmol/L, IQR	1.5 (0.7–4.3)	1.2 (0.5–2.3)	3.7 (0.7–8.0)	0.245
Primary lung disease, *n* (%)	24 (82.8)	16 (80.0)	8 (88.9)	0.558
**Comorbidity**				
Leucocythemia, *n* (%)	2 (6.9)	1 (5.0)	1 (11.1)	0.548
Tumor, *n* (%)	2 (6.9)	0 (0)	2 (22.2)	0.029
Autoimmune system diseases, *n* (%)	1 (3.4)	1 (5.0)	0 (0)	0.495
Neuromuscular disease, *n* (%)	1 (3.4)	1 (5.0)	0 (0)	0.495
**Complication**				
Shock, *n* (%)	22 (75.9)	13 (65.0)	9 (100.0)	0.042
AKI, *n* (%)	16 (55.2)	8 (40.0)	8 (88.9)	0.014
Hepatic dysfunction, *n* (%)	9 (31.0)	5 (25.0)	4 (44.4)	0.295
Gastrointestinal dysfunction, *n* (%)	6 (20.7)	3 (15.0)	3 (33.3)	0.260
MODS, *n* (%)	15 (51.7)	8 (40.0)	7 (77.8)	0.060
**Baseline at ECMO initiation**				
LUS score, IQR	28 (27–30)	28 (27–30)	29 (28–30)	0.427
PaO_2_/FiO_2_, mmHg, IQR	60 (55–68)	63 (56–68)	60 (51–64)	0.227
OI, IQR	32 (28–41)	31 (27–37)	41 (32–43)	0.073
PaCO_2_, mmHg, IQR	52 (43–65)	52 (44–60)	61 (34–75)	0.533
Cdyn, ml/cmH_2_O.kg, IQR	0.33 (0.30–0.39)	0.34 (0.30–0.40)	0.33 (0.30–0.38)	0.758
**Mechanical ventilation settings**				
PIP, cmH_2_O, IQR	29 (28–32)	30 (28–32)	29 (28–31)	0.651
PEEP, cmH_2_O, IQR	12 (10–13)	13 (12–14)	13 (12–15)	0.278
FiO_2_, %, IQR	100 (100–100)	100 (100–100)	100 (100–100)	0.334
**Mode of ECMO**				
VA-ECMO, *n* (%)	21 (72.4)	13 (65.0)	8 (88.9)	0.183
VV-ECMO, *n* (%)	8 (27.6)	7 (35.0)	1 (11.1)	0.183

*LUS, lung ultrasound; ECMO, extracorporeal membrane oxygenation; VA-ECMO, veno-arterial extracorporeal membrane oxygenation; VV-ECMO, veno-venous extracorporeal membrane oxygenation; PaO_2_/FiO_2_, PaO_2_:FiO_2_ ratio; PEEP, positive end-expiratory pressure; AKI, acute kidney injury; MODS, multiple organ dysfunction syndrome; PIP, positive inspiration pressure; Cdyn, dynamic lung compliance; PRISM III, pediatric risk mortality III; FiO_2_, fractional concentration of oxygen in inspired gas; BMI, body mass index.*

### Lung Ultrasound Score, Respiratory System Compliance, and Daily Fluid Balance During Extracorporeal Membrane Oxygenation Between Survivors and Non-survivors

There were no differences about the values of LUS-0 h, LUS-24 h, and LUS-48 h between survivors and non-survivors (all *P* > 0.05); however, the values of LUS-72 h and LUS-wean were significantly lower in survivors than in non-survivors (both *P* < 0.001) (Table 2). In addition, the degree of negative fluid balance of the 2nd day (48–24 h) or 3rd day (72–48 h) after ECMO support was greater in survivors than in non-survivors (*P* = 0.032 and *P* = 0.007, respectively) (Table 2).

**TABLE 2:** Comparison of LUS score, respiratory compliance, and daily fluid balance during ECMO between survivors and non-survivors (IQR).

Parameters	Survivors (*n* = 20)	Non-survivors (*n* = 9)	*P-value*
**LUS score**			
0 h	28 (27–30)	29 (28–30)	0.427
24 h	31 (29–31)	31 (30–32)	0.219
48 h	27 (26–28)	28 (27–30)	0.101
72 h	22 (20–23)	30 (25–31)	<0.001
wean	12 (10–15)	30 (28–34)	<0.001
**Cdyn, ml/kg.cmH_2_O**			
0 h	0.38 (0.30–0.40)	0.35 (0.32–0.37)	0.366
24 h	0.32 (0.27–0.35)	0.30 (0.27–0.32)	0.380
48 h	0.35 (0.30–0.38)	0.31 (0.29–0.36)	0.256
72 h	0.44 (0.41–0.55)	0.35 (0.28–0.38)	<0.001
wean	0.66 (0.63–0.70)	0.26 (0.16–0.32)	<0.001
**Required ECMO blood flow, ml/kg.min**			
0 h	87 (74–92)	83 (74–108)	0.799
24 h	76 (65–86)	90 (62–104)	0.285
48 h	80 (63–103)	97 (52–132)	0.524
72 h	62 (43–76)	85 (74–111)	0.008
wean	11 (8–29)	78 (12–135)	0.023
**Required ECMO gas flow, L/min**			
0 h	2.0 (1.3–2.0)	1.8 (1.0–3.1)	0.481
24 h	1.4 (1.0–2.0)	1.6 (0.8–3.1)	0.609
48 h	1.7 (0.9–2.0)	1.3 (0.8–3.4)	0.759
72 h	1.5 (1.0–2.0)	1.3 (0.6–3.9)	0.979
wean	0 (0–0.4)	1.8 (0–2.5)	0.042
**Daily fluid balance volume, ml/kg.d**			
24–0 h	17 (12–22)	21 (17–30)	0.202
48–24 h	–16[–18–(–12)]	–11[–12–(–8)]	0.032
72–48 h	–12[–14–(–9)]	–6[–9–(–4)]	0.007

*LUS, lung ultrasound; ECMO, extracorporeal membrane oxygenation; Cdyn, dynamic lung compliance.*

The value of LUS-0 h was 28 (IQR, 27–30), and then gradually decreased to 22 (IQR, 20–23) of LUS-72 h in the survival group (*P* < 0.001), but there was no significant decrease in the non-survival group (Table 2). In addition, the value of Cdyn was 0.35 (IQR, 0.30–0.38) ml/cm.H_2_O.kg at 48 h after ECMO support, then gradually increased to 0.66 (IQR, 0.63–0.70) ml/cm.H_2_O.kg when weaned from ECMO in the survival group (*P* < 0.001). However, the change of Cdyn was gradually decreased from 0.31 (IQR, 0.29–0.36) ml/cm.H_2_O.kg of LUS-48 h to 0.26 (IQR, 0.16–0.32) ml/cm.H_2_O.kg of LUS-wean in the non-survival group (*P* = 0.012) (Table 2). Moreover, both required blood flow and required gas flow had significant reduction from 48 h after ECMO support to ECMO weaning in the survival group (Table 2).

**FIGURE 2:**
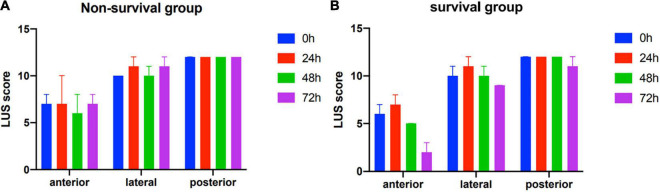
Zone distribution of various lung ultrasound findings in non-survivors **(A)** and survivors **(B)**.

Lung ultrasound scores of all the distribution regions during the first 72 h of ECMO support were not significantly decreased in the non-survival group (Figure 2A). In the survival group, the LUS scores of bilateral anterior, lateral, and posterior regions were all significantly improved during the first 72 h of ECMO support (all *P* < 0.01). Moreover, all the peak values of LUS score in anterior, lateral, and posterior regions were displayed at 24 h after ECMO support, then the values of the LUS score in the anterior and lateral regions, but not posterior, were decreased in the survival group (*P* < 0.05, Figure 2B).

### Correlation of Lung Ultrasound Score, Respiratory Dynamics, and Fluid Balance Volume

**FIGURE 3:**
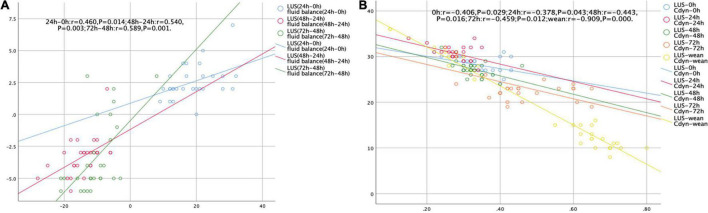
Scatterplots demonstrating the correlation between LUS score and daily fluid balance volume **(A)** and Cdyn **(B)**.

An excellent correlation was observed between LUS score and daily fluid balance during the first 72 h of ECMO treatment (all *P* < 0.05) (Figure 3A). In addition, LUS score was negatively correlated with Cdyn during ECMO support (all *P* < 0.05) (Figure 3B).

### Receiver Operating Characteristic Analysis of Lung Ultrasound Score for Prediction of Prognosis in Patients With Pediatric Acute Respiratory Distress Syndrome

Receiver operating characteristic analysis of significantly changed variables during ECMO support was constructed for predicting PICU mortality, and the value of AUC for LUS-72 h, 3rd day fluid balance-(72–48 h), required ECMO blood flow-72 h, or Cdyn-72 h was 1.000 [95% *CI*: 1.000–1.000], 0.831 [95% *CI*: 0.659–1.000], 0.825 [95% *CI*: 0.653–0.997], or 0.913 [95% *CI*: 0.748–1.000], respectively (all *P* < 0.05). Among them, the cutoff value of LUS-72 h for predicting PICU mortality was 24 with a sensitivity of 100% and a specificity of 100%, respectively (Table 3).

**TABLE 3:** Receiver operating characteristic analysis of variables for predicting PICU mortality.

Parameters	*AUC*	95% *CI*	*Cutoff*	*Sensitivity* *(%)*	*Specificity* *(%)*	*P-value*
LUS-72 h	1.000	(1.000–1.000)	24	100.0	100.0	0.000
Fluid balance-(48–24 h)	0.763	(0.532–0.993)	–13	87.5	75.0	0.033
Fluid balance-(72–48 h)	0.831	(0.659–1.000)	–7	75.0	90.0	0.007
Required ECMO blood flow -72 h (ml/min)	0.825	(0.653–0.997)	76.3	62.5	80.0	0.008
1/Cdyn-72 h	0.913	(0.748–1.000)	2.74	87.5	100.0	0.001

*LUS, lung ultrasound; ECMO, extracorporeal membrane oxygenation; Cdyn, dynamic lung compliance; PICU, pediatric intensive care unit; CI, confidence interval; ROC, receiver operating characteristic; AUC, area under the curve.*

### Comparison of Outcome and Complications in Patients With Lung Ultrasound-72 h ≥ 24 or Lung Ultrasound-72 h < 24

Patients were stratified in LUS-72 h ≥ 24 group and LUS score-72 h < 24 group. Patients with LUS-72 h ≥ 24 had significantly higher PICU mortality and greater length of PICU stay than patients with LUS-72 h < 24 [100% (9/9) vs. 0% (0/20), *P* < 0.001]. During ECMO support, the proportion of patients requiring CRRT as an adjuvant therapy to maintain fluid balance was higher in LUS-72 h ≥ 24 group than LUS-72 h < 24 group (Table 4). There were 9 dead patients who were all with LUS-72 h ≥ 24. Among these 9 dead cases, inflammatory lung collapse (3 cases), multiorgan failure (3 cases), acute liver failure (1 patient), intracranial hemorrhage (1 case), and refractory septic shock (1 case) were the causes of death.

**TABLE 4:** Description of clinical outcomes and complications between LUS-72 h ≥ 24 and LUS-72 h < 24.

Outcomes	Total (*n* = 29)	LUS-72 h ≥ 24 (*n* = 9)	LUS-72 h < 24 (*n* = 20)	*P-value*
Duration of ECMO, hours, IQR	162 (86–273)	161 (76–367)	179 (118–275)	0.671
Duration of ventilation, hours, IQR	286 (149–434)	250 (104–611)	288 (164–438)	0.604
Length of PICU stay, days, IQR	15 (12–34)	11 (6–26)	19 (14–35)	0.042
PICU mortality, *n* (%)	9 (31.0)	9 (100.0)	0 (0)	<0.001
Shock, *n* (%)	16 (55.2)	9 (100.0)	7 (35.0)	0.001
Need of CRRT, *n* (%)	22 (75.9)	9 (100.0)	13 (65.0)	0.042
VIS, IQR	150 (63–180)	190 (135–230)	115 (10–170)	0.015
DIC, *n* (%)	6 (20.7)	3 (33.3)	3 (15.0)	0.260
Secondary infections, *n* (%)	1 (3.4)	0 (0)	1 (5.0)	0.495
Hemolysis, *n* (%)	6 (20.7)	3 (33.3)	3 (15.0)	0.260
Thrombosis, *n* (%)	1 (3.4)	0 (0)	1 (5.0)	0.495
Intracranial hemorrhage, *n* (%)	3 (10.3)	2 (22.2)	1 (5.0)	0.159
Gangrene of limbs, *n* (%)	1 (3.4)	0 (0)	1 (5.0)	0.495
MODS, *n* (%)	19 (65.5)	7 (77.9)	12 (60.0)	0.351

*LUS, lung ultrasound; ECMO, extracorporeal membrane oxygenation; PICU, pediatric intensive care unit; CRRT, continuous renal replacement therapy; VIS, vasoactive index score; DIC, disseminated intravascular coagulation; MODS, multiple organ dysfunction syndrome.*

## Discussion

Lung monitoring is crucial for evaluation of treatment effectiveness and early identification of complications in patients with ARDS (16). In this prospective cohort study, we monitored the changes of LUS score in 29 patients with pARDS during ECMO support, and the main findings are as follows: (1) patients with LUS-72 h ≥ 24 have a higher risk of PICU mortality; and (2) LUS score is correlated to daily fluid balance volume and the value of Cdyn. These results give us new insight into the tool for monitoring the severity of pulmonary edema during ECMO support, and it is helpful for assessing the process of pARDS and the recovery of lung function. A high LUS score at 72 h after ECMO support might be associated with a worse outcome.

Lung ultrasound is an increasingly used tool for monitoring pulmonary lesions or improving the diagnosis of pneumonia in critically ill children (17–19). The area of loss of aeration can be distinguished and quantified as LUS score by examining 12 thoracic areas; each area’s score ranges from 0 (normal aeration) to 3 (complete loss of aeration), depending on the visualized lines (18). The LUS score shows the sum of each area’s score and ranges from 0 to 36, providing the semi-quantification of lung conditions (17). Increased EVLW leads to impaired gas exchange contributing to respiratory failure in ARDS. LUS score is a well-known non-invasive and repeatable method to assess EVLW in patients with ARDS (20, 21), and LUS score is well correlated to the oxygenation status in both neonates and children (9, 15). Systematic prospective multi-institutional studies indicated that EVLW is directly correlated to PaO_2_/FiO_2_ and OI (22–24). Moreover, a strong negative association between LUS score and Cdyn at 48 h, Day 5 and Day 10 of commencement of VV-ECMO was observed in adult patients with ARDS (25). Consistently, our study revealed that LUS score mildly increased within 24 h, then gradually decreased after 72 h ECMO support in survivors. Importantly, LUS score was negatively correlated to Cdyn at 24, 48, and 72 h after ECMO support in children with pARDS. To the best of our knowledge, this is the first report to elucidate the changes of LUS score and its role in predicting the outcome of patients with severe pARDS during ECMO support.

The initiation of ECMO might trigger a systemic inflammation, which is involved in the activation of coagulation cascade and complement systems, and endothelial injury (26, 27), thus potentially contributing to lung edema. In the present study, the mild increasing LUS score was observed in early ECMO support (within 24 h), which could be related to the temporary deterioration inflammatory response by extracorporeal life support. Fluid balance is associated with the outcome of patients on ECMO. Lee et al. (28) reported that a higher positive cumulative fluid balance on Day 3 of ECMO was associated with increased 28-day mortality. In the present study, daily fluid balance during the first 3 days of ECMO support was significantly correlated to LUS score, and LUS score ≥ 24 after 72 h ECMO support was associated with a worse outcome in patients with pARDS.

According to a retrospective analysis of 2727 pediatric ECMO runs reported to the ELSO registry from 2013 to 2017, OI and duration of mechanical ventilation before ECMO deployment were both independently associated with in-hospital mortality (29). Duration of mechanical ventilation could be closely related to the recovery of pulmonary function. Prat et al. reported that regional changes of LUS could be a tool in prediction of prone positioning oxygenation response in ARDS patients (30). In our study, LUS score was correlated with Cydn-72. Intriguingly, LUS score was used in predicting the outcomes of ARDS caused by COVID-19 (10, 31, 32). In the present study, the LUS scores of posterior regions were highest and improved slowest after ECMO support in the survival group. More importantly, LUS-72 h for predicting PICU mortality is superior to fluid balance-(3rd day, 72–48 h) and Cdyn-72 h. All these results suggest that assessing regional fluid management in the lung could be more important than global fluid management in children under ECMO support. Our results revealed that patients with LUS-72 h ≥ 24 had a higher risk of PICU mortality and longer PICU stay days.

## Study Limitations

There were several limitations of this study. First, this is a single center study with limited sample size and children aged ≤ 14 years old, and selection bias cannot be ruled out. Second, the lack of LUS scores from 72 h to ECMO weaning is another limitation. Third, we did not analyze the relationship between the LUS score and other methods for determining EVLW like PiCCO_2_ due to the difficulty for carrying out PiCCO_2_ in pediatric patients. Fourth, the value of LUS score in pediatric patients with ARDS without ECMO was lacking, the potential role of the LUS score in assessing the severity of pediatric ARDS needs further well-designed clinical research. Fifth, the sub-group analysis according to whether patients were in a prone position was not performed, which could be a more interesting clinical research issue. Nevertheless, LUS is a convenient and non-invasive tool for lung monitoring in patients with pARDS under ECMO support. Importantly, LUS-72 h score is significantly associated with PICU mortality, which could be a potential parameter for guiding the management of lung function in these patients. Our findings are worth further investigation to validate the conclusion in a well-designed multicenter study with a larger population.

## Conclusion

Lung ultrasound score is correlated to daily fluid balance volume and Cdyn. The LUS score ≥ 24 at 72 h of ECMO support is associated with a worse outcome of pARDS. LUS can provide a promising tool for lung monitoring in patients with ARDS receiving ECMO.

## Data Availability Statement

The original contributions presented in the study are included in the article/Supplementary Material, further inquiries can be directed to the corresponding author.

## Ethics Statement

This study was approved by the Ethics Committee of Shanghai Children’s Hospital, Shanghai Jiao Tong University, China (Approval number: 2016R011-E02). All patients’ parents or relatives signed informed consent.

## Author Contributions

YuZ, CW, and FW contributed in writing the manuscript. FW, YiZ, JS, JD, TS, and YS analyzed the patients’ data. FW performed the lung ultrasound and collected the patients’ data. YuZ was responsible for the design of this study. All authors read and approved the final manuscript.

## Conflict of Interest

The authors declare that the research was conducted in the absence of any commercial or financial relationships that could be construed as a potential conflict of interest.

## Publisher’s Note

All claims expressed in this article are solely those of the authors and do not necessarily represent those of their affiliated organizations, or those of the publisher, the editors and the reviewers. Any product that may be evaluated in this article, or claim that may be made by its manufacturer, is not guaranteed or endorsed by the publisher.

## Supplementary Material

Supplementary Figure 1 Partition of chest wall in lung ultrasound examination protocol.

## Abbreviations

AKI: acute kidney injury
AUC: area under the curve
BMI: body mass index
Cdyn: dynamic lung compliance
CI: confidence interval
CRRT: continuous renal replacement therapy
CT: computed tomography
DIC: disseminated intravascular coagulation
ECMO: extracorporeal membrane oxygenation
ELSO: extracorporeal life support organization
EVLW: extravascular lung water
FiO_2_: fractional concentration of oxygen in inspired gas
LUS: lung ultrasound
MAP: mean airway pressure
MODS: multiple organ dysfunction syndrome
OI: oxygen index
PF ratio: PaO_2_/FiO_2_ ratio
pARDS: pediatric acute respiratory distress syndrome
PEEP: positive end-expiratory pressure
PALICC: The Pediatric Acute Lung Injury Consensus Conference
PiCCO_2_: pulse indicator continuous cardiac output
PICU: pediatric intensive care unit
PIP: positive inspiration pressure
PRISM III: pediatric risk mortality III
ROC: receiver operating characteristic
VA-ECMO: veno-arterial extracorporeal membrane oxygenation
VV-ECMO: veno-venous extracorporeal membrane oxygenation
VIS: vasoactive index score.
