# Supplementary figures and images for: Lung Ultrasound in Pediatric Acute Respiratory Distress Syndrome Received Extracorporeal Membrane Oxygenation: A Prospective Cohort Study
Source: Front Pediatr. 2022 Mar 28;10:798855. doi: 10.3389/fped.2022.798855 (PMC8995848; doi:10.3389/fped.2022.798855)

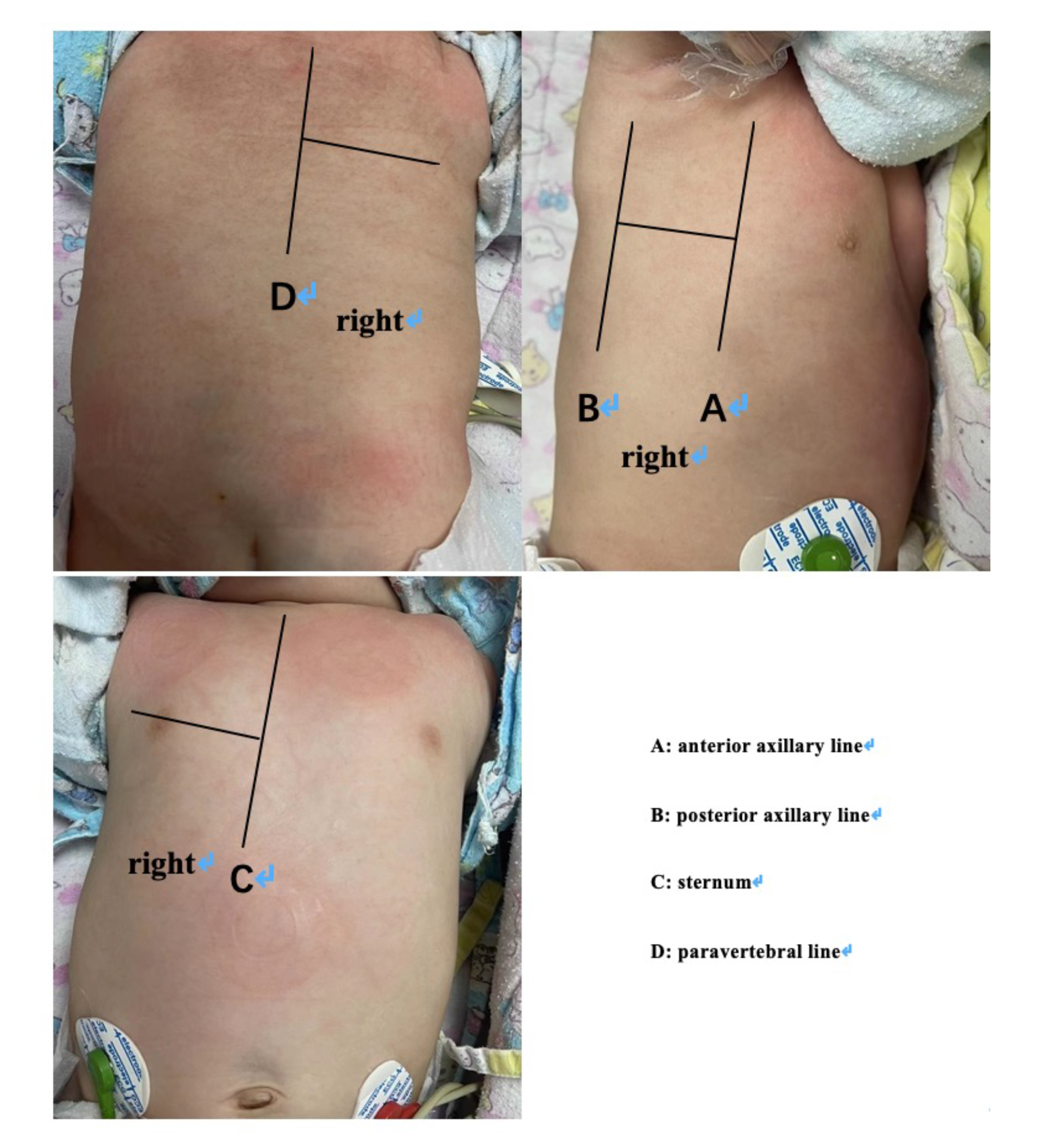

Supplement: Supplementary Figure 1 — Partition of chest wall in lung ultrasound examination protocol. [file Image_1.TIFF]
